# Supplementary material for: Vitamin D Modified DSS-Induced Colitis in Mice via STING Signaling Pathway
Source: Biology (Basel). 2025 Jun 18;14(6):715. doi: 10.3390/biology14060715 (PMC12190092; doi:10.3390/biology14060715)
Supplement: Supplementary file 1 [file biology-14-00715-s001.zip › Supplementary Materials S1: 16s-report of gut microbiota/Groups/diff_analysis/UnivarTestKEGG/figure/barplot/top10log.pdf]

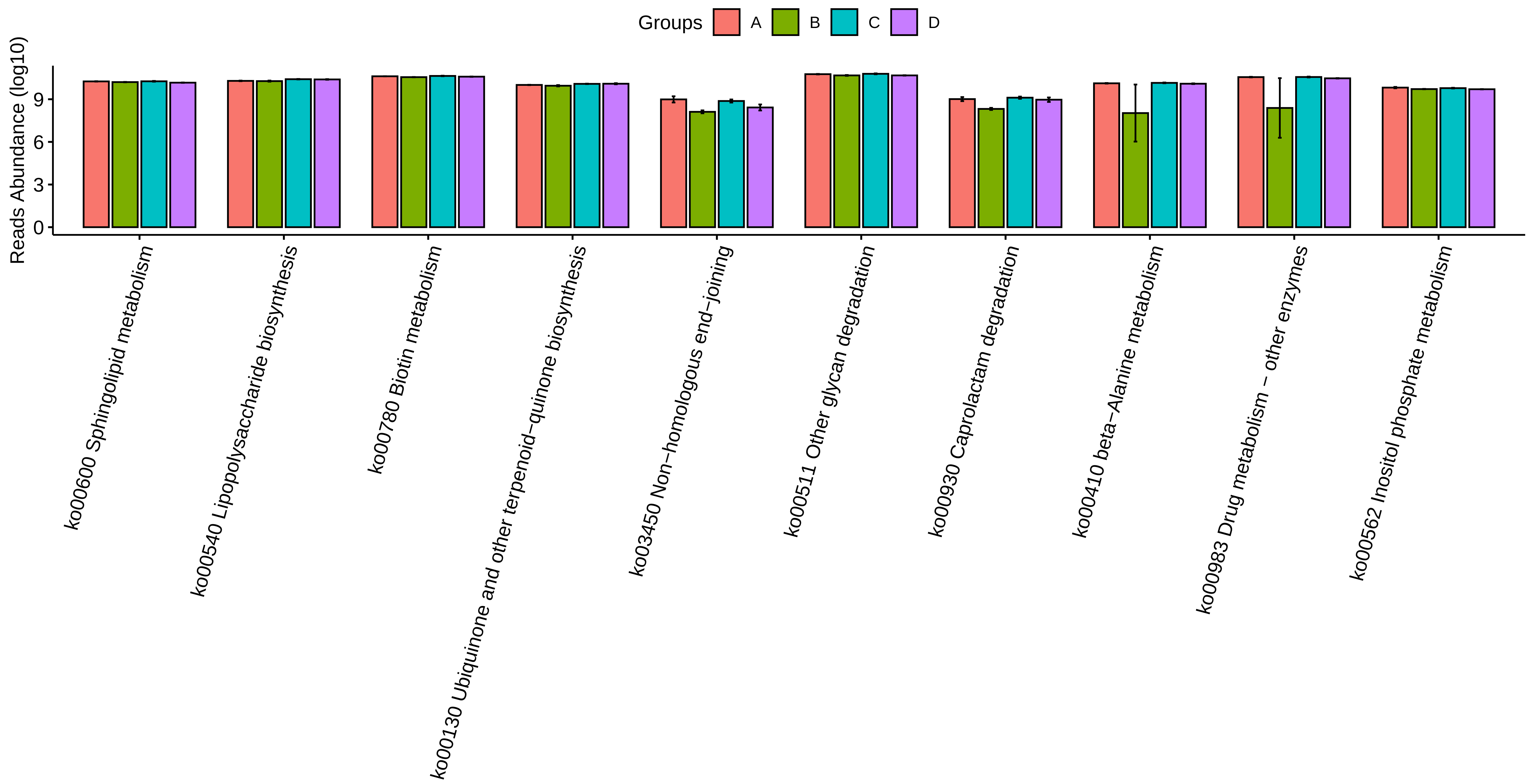

Groups
A
B
C
D
Reads Abundance (log10)
0
3
6
9
ko00600 Sphingolipid metabolism
ko00540 Lipopolysaccharide biosynthesis
ko00780 Biotin metabolism
ko00130 Ubiquinone and other terpenoid−quinone biosynthesis
ko03450 Non−homologous end−joining
ko00511 Other glycan degradation
ko00930 Caprolactam degradation
ko00410 beta−Alanine metabolism
ko00983 Drug metabolism − other enzymes
ko00562 Inositol phosphate metabolism
